# The SAP130/Mincle axis was involved in sevoflurane-induced neuronal death and microglial activation in juvenile mice

**DOI:** 10.3389/fphar.2025.1647329

**Published:** 2025-07-28

**Authors:** Zi-Heng Zhou, Xiao-Xiang Chen, Wen Zhang, Bin Shu, Ying Chen

**Affiliations:** ^1^Department of Burn, Wound Repair and Reconstruction, The First Affiliated Hospital, Sun Yat-sen University, Guangzhou, China; ^2^Department of Anesthesiology, The First Affiliated Hospital, Sun Yat-sen University, Guangzhou, China

**Keywords:** microglia, neuroinflammation, neuron, SAP130, Mincle, sevoflurane

## Abstract

**Introduction:**

Sevoflurane is widely used in pediatric anesthesia and has raised concerns for years regarding its neurotoxic effects on the developing brain. Studies have shown that sevoflurane can lead to neuronal cell death and neuroinflammation, which further contribute to sevoflurane-induced neurotoxicity manifested as delirium or cognitive deficits. However, the molecular mechanism remains poorly understood. A factor of interest is Sin3A-associated protein 130 (SAP130), which can be released by dead or damaged cells and trigger sterile inflammation, exacerbating tissue damage by activating the macrophage-inducible C-type lectin (Mincle) receptor. However, whether the SAP130/Mincle axis is involved in sevoflurane-induced neurotoxicity remains unknown.

**Methods:**

Using a young murine sevoflurane exposure model and a primary neuron–microglia co-culture system, we examined changes in neuronal cell death, microglial activation, cytokine production, and the expression levels of SAP130- and Mincle-signaling-associated proteins after sevoflurane exposure. We then applied SAP130-neutralizing antibody and the Syk inhibitor piceatannol to assess the impact of inhibiting the Mincle pathway on microglial activation and sevoflurane-induced neurotoxicity.

**Results:**

The results demonstrated that sevoflurane exposure increased the number of dead neurons with SAP130 upregulation and induced microglial activation with cytokine production in the hippocampus. These changes occurred only in the neuron–microglia co‐culture system but not in neuron or microglia monoculture. Neutralizing SAP130 or pharmacologically inhibiting syk diminished microglial activation and neuronal cell death by suppressing the SAP130/Mincle signaling pathway.

**Discussion:**

These findings suggest that the SAP130/Mincle axis plays a crucial role in neuronal death and microglial activation in sevoflurane-induced neurotoxicity. Targeting this axis emerges as a potential therapeutic strategy to mitigate the neurotoxic effects of sevoflurane.

## Introduction

Sevoflurane is one of the most commonly used inhaled anesthetics, especially for pediatric anesthesia. As such, concerns regarding its potential neurotoxic effects on the developing brain have persisted for decades. Extensive preclinical evidence indicates that exposure to sevoflurane or other general anesthetic agents could induce neuronal cell death, neuroinflammation, and even cognitive deficits in young rodents or non-human primates (McCann and Soriano, 2019; Liu et al., 2020). Although clinical studies have not consistently shown a direct link between general anesthetic exposure and long-term neurodevelopmental impairment or cognitive dysfunction in humans (McCann and Soriano, 2019; McCann et al., 2019; Davidson and Sun, 2018), recent clinical data show that general anesthesia may contribute to postoperative delirium and other cognitive abnormalities in children, and the incidence of children anesthetized by sevoflurane ranges from 40% to 80%, which is significantly higher than that in adults (Urits et al., 2020; Shi et al., 2019; Yang et al., 2022). Furthermore, sevoflurane has been associated with a higher incidence of cognitive abnormalities than other intravenous anesthetics, like propofol (Urits et al., 2020). These findings raise concerns about the acute neurotoxic effects of sevoflurane. However, the underlying mechanism remains unclear.

Neuronal cell death was one of the leading causes of sevoflurane-induced neurotoxicity that contributes to cognitive impairment, and apoptosis was regarded as the main pathway leading to neuronal cell death (Sun et al., 2022). Various research studies have demonstrated that prolonged sevoflurane exposure can trigger both the intrinsic and extrinsic apoptotic pathways in the brain, with the developing and aged brain being more vulnerable to its neurotoxicity (Sun et al., 2022; Zhao et al., 2023; Xu et al., 2023b; Areias et al., 2023). In addition, recent studies reported that sevoflurane can induce neuronal necroptosis in both neonatal and aged brains (Liu et al., 2024; Xu et al., 2022; Zhang et al., 2024b). Interestingly, neuroinflammation appears to be a concomitant response to sevoflurane-induced neuronal cell death (Zhang et al., 2024b; Sun et al., 2015; Zhao et al., 2023). For example, sevoflurane-induced neuronal apoptosis and cytokine elevation in the hippocampus, with both of these effects being attenuated by the knockdown of lncRNA HOXA11-AS or intravenous injection of bone marrow stromal cells (Zhao et al., 2023; Sun et al., 2015). Another study indicated that microglia M1 polarization was involved in sevoflurane-induced necroptosis (Zhang et al., 2024b). However, the interaction between sevoflurane-induced neuronal cell death and neuroinflammation needs further investigation.

Neuroinflammation has been regarded as a key contributor to the mechanism of perioperative neurocognitive disorders, including anesthesia-related cognitive dysfunctions (Alam et al., 2018; Li et al., 2022; Liu et al., 2023b; Luo et al., 2019; Xu et al., 2023a). Our previous study also reported that isoflurane-induced NLRP3 activation contributes to cognitive impairment in aged mice (Wang et al., 2018). As for sevoflurane-induced neurotoxicity, neuroinflammation is also regarded as a pivotal factor in sevoflurane-induced cognitive impairment. Various studies have demonstrated that sevoflurane-enhanced neuroinflammation triggers cognitive dysfunction in rodent models through different molecular pathways (Zhu et al., 2017; Tang et al., 2021). Prolonged sevoflurane exposure could also induce complement cascades and microglial activation that contributed to synaptic elimination and cognitive dysfunction (Xu et al., 2023a). Microglia depletion could attenuate sevoflurane-related synaptic loss and cognitive impairment (Xu et al., 2023a). Moreover, another study using a microglia–neuron co-culture system demonstrated that activated microglia mediated neuronal apoptosis (Xiao et al., 2023). These findings, combined with others, indicate that activated microglia play a contributory role in sevoflurane-induced neuroinflammation and cognitive dysfunction as microglia are an important source of inflammatory factors in the brain, mediate inflammatory responses, and can even exacerbate neuronal damage or death (Huang et al., 2021; Wang et al., 2021; Dai et al., 2021; Gao et al., 2024). However, the precise mechanisms underlying sevoflurane-triggered microglial activation and its involvement in neurotoxicity remain poorly understood.

Damage-associated molecular patterns (DAMPs) are endogenous molecules released from damaged or dead cells due to various types of cell death, such as necrosis, apoptosis, pyroptosis, ferroptosis, and NETosis (Huang et al., 2024; Ma et al., 2024; Murao et al., 2021). DAMPs contain multiple types of molecules—including nucleic acids, proteins, ions, glycans, and metabolites—that can be recognized by innate immune receptors to trigger inflammatory responses (Huang et al., 2024; Ma et al., 2024). Thus, DAMPs play crucial roles in inflammation and related diseases. Sin3A associated protein 130 (SAP130), a subunit of the histone deacetylase complex, is a type of DAMP (Drouin et al., 2020; Williams, 2017). SAP130 can be released from dead or damaged cells, triggering sterile inflammation that further aggravates tissue damage (Drouin et al., 2020; Lv et al., 2021; Zhang et al., 2024a). Macrophage-inducible C-type lectin (Mincle) recognizes SAP130 and other ligands, including β-glucosylceramide, cholesterol, and glycolipids found in bacteria and fungi (Lu et al., 2018). Upon binding to its ligand, such as SAP130, Mincle recruits and phosphorylates its adaptor protein syk to elevate the production of inflammatory factors (Drouin et al., 2020; Huang et al., 2023; Williams, 2017; Del Fresno et al., 2018). In the central nervous system, Mincle is mainly expressed on microglia and plays a significant role in the progression of brain injury. Activation of the Mincle signaling pathway through its ligands, such as SAP130 or properdin, facilitated the progression of ischemic stroke, and pharmacological inhibition or knockdown of Mincle signaling can mitigate damage (Suzuki et al., 2013; Liu et al., 2023a). Similarly, neutralization of SAP130 or blockage of the Mincle/syk pathway by BAY61-3606 attenuated neuroinflammation and neurological deficits in the traumatic brain injury animal model, highlighting the contribution of Mincle in the pathology of traumatic brain injury (He et al., 2022; de Rivero Vaccari et al., 2015). Furthermore, recent studies suggested that the Mincle receptor is a contributor to the development of multiple sclerosis by mediating neuroinflammation via recruiting T-cell infiltration and activation (N'Diaye et al., 2020). However, the role of the SAP130/Mincle signaling pathway in other neurological disorders, including anesthesia-related neurotoxicity, remains unclear. Given neuronal cell death and neuroinflammation caused by sevoflurane, we hypothesize that sevoflurane-induced neuronal cell death or damage resulted in SAP130 release, which triggered the Mincle signaling pathway and promoted microglial activation with cytokine production. This cascade may, in turn, aggravate neuronal cell death. To test this hypothesis, we evaluated the changes in SAP130 and cytokines, Mincle signaling, microglia activation, and neuronal cell death in the sevoflurane-exposed brain and the primary neuron–microglia co-culture system. We further assessed the effects of SAP130 neutralization or Mincle signaling inhibition on the post-anesthetic brain and the neuron–microglia co-culture system.

## Materials and methods

### Ethic declaration

All animal care and uses were carried out in accordance with the Guide for the Care and Use of Laboratory Animals, and all the procedures were approved by the Animal Ethics Committee of Sun Yat-sen University (SYSU-IACUC-2025-000954). Postnatal 21-day (P21) male C57BL/6 wild-type (WT) mice and postnatal 1-day mice obtained from the Laboratory Animal Center of Sun Yat-sen University were used in this study. All mice were housed in a temperature-controlled room under a 12/12 h light/dark cycle and were provided free access to food and water.

### Animal treatment

Mice were exposed to 3.5% sevoflurane anesthesia with oxygen for 2 h in a chamber equipped with an air inlet and outlet, along with an exhaust gas reclaim system to maintain a stable anesthesia concentration. The control group received oxygen only for 2 h under the same conditions. This protocol was performed as previously described with some modifications (Rapido et al., 2025).

The mice received an intraperitoneal injection of the syk-specific inhibitor piceatannol at a dose of 20 mg/kg 1 h before sevoflurane anesthesia. The same volume of vehicle was given to the mice as the control.

### Primary neuronal culture

Primary neuron culture was performed as previously reported (Ahlemeyer and Baumgart-Vogt, 2005). In brief, cerebral cortices were collected from postnatal 1-day mice and dissected in Hank’s solution after removal of the meninges and vascular membranes. They were digested in 3 mL of papain (Melone/China, 2 mg/mL) containing DNase I (Sigma, Germany, 5 kU/mL) at 37°C for 30 min, and digestion was terminated by adding 1 mL of fetal bovine serum (FBS, Gibco, New York, United States). After centrifugation at 1,000 rpm for 5 min, the pellet was collected and resuspended in culture medium with 10% FBS. After gentle pipetting, the cell suspension was collected and seeded on poly-L-lysine-coated 6-well plates. After 6–8 h of incubation, the medium was replaced with Neurobasal-A (Gibco) with 2% B27 and changed every 3 days. After 7 days, neuron–microglia co-culture systems were established by inserting microglia-containing Transwell inserts.

### Primary microglia culture

To prepare primary microglia cultures, cortices from 1-day-old mice were harvested on ice. After the removal of meninges and vascular membranes, brain tissues were dissected in Hank’s solution and digested in 0.125% trypsin (Gibco, 2 mg/mL) and DNase I (Sigma, 5 kU/mL) for 20 min at 37°C. Pooled cells prepared from two mice were planted in 75 cm^2^ cell culture flasks for 14 days. After incubation with 0.3% lidocaine for 10 min followed by gentle shaking, primary microglia were detached from the mixed glial culture. The floating microglial cells were collected and seeded in a 0.2-μm transwell chamber. After 24 h, a microglia-containing transwell was used to establish the neuron–micro co-culture system.

### Cell treatment

Primary neurons or microglia were exposed to 3.5% sevoflurane, 21% O_2_, and 5% CO_2_ at 37°C for 6 h in a special sealed chamber, following the previous protocol with some modification (Rapido et al., 2025). Sevoflurane concentration was maintained at 3.5% ± 0.2% during the intervention. The control group received 21% O_2_ and 5% CO_2_ under the same conditions. Primary neuron–microglia co-cultured systems were subjected to the specific syk inhibitor, piceatannol (25 µM) or the neutralized SAP130 antibody (1 µg/mL) 30 min before sevoflurane exposure and were then incubated with sevoflurane for another 6 h. The same volume of vehicle was administered to the cells as the control. Primary microglia were treated with lipopolysaccharide (LPS) at a concentration of 1 µg/mL for 24 h as a positive control.

### CCK-8 assay

Primary neurons or microglia were seeded into 96-well plates for 24 h and exposed to 3.5% sevoflurane for 6 h. Cells were incubated with CCK-8 working solution for 4 h at 37°C. The OD values at 450 mm were measured using a microplate reader. Cell viability (%) = (OD_sev_−OD_blank_)/ (OD_con_−OD_blank_) ×100%.

### Hoechst/PI staining

Twenty-four hours after sevoflurane treatment, primary neurons cultured with or without microglia were rinsed with PBS and incubated in Hoechst and PI working solution (Beyotime, China; 1:200) at 4°C for 30 min. After rinsing with PBS, microphotographs were visualized under a fluorescent microscope.

### Enzyme-linked immunosorbent assay

The cell supernatants after sevoflurane treatment were collected. SAP130 and cytokines including IL-1β, IL-6, and TNF-α were detected using an SAP130 ELISA kit (Cusabio, China) and IL-1β, IL-6, and TNF- α ELISA kits (R&D, MN, United States), respectively, according to the manufacturer’s instructions.

### RNA isolation and real-time PCR assay

Cultured primary microglia were harvested at the end of the experiment. Total mRNA was isolated using RNAiso Plus (TaKaRa, Japan). Only mRNA samples with an OD260/280 ratio between 1.8 and 2.0 were qualified to be converted into complementary DNA (cDNA) using the PrimeScript^TM^ Master Mix Kit (TaKaRa, Japan). The expression of targeted genes was assessed through real-time PCR with their specific primers:

GAPDH-F: 5′-GGT GAA GGT CGG TGT GAA CG-3′

GAPDH-R: 5′-CTC GCT CCT GGA AGA TGG TG-3′,

IL-1β-F: 5′-AGA GCC CAT CCT CTG TGA CT-3′

IL-1β-R: 5′-GGA GCC TGT AGT GCA GTT GT-3′,

IL-6-F: 5′-CCC AAT TTC CAA TGC TCT CCT-3′

IL-6-R: 5′-CGC ACT AGG TTT GCC GAG TA-3′,

TNF-α-F: 5′-ATG GCC TCC CTC TCA TCA GT-3′

TNF-α-R: 5′-TTT GCT ACG ACG TGG GCT AC-3′.

### Western blotting

The harvested primary neurons or microglia were subjected to Western blot analysis. The protein was extracted using RIPA lysis buffer containing protease and phosphatase inhibitors. The total lysate was collected, subjected to 8%–12% SDS-PAGE gel electrophoresis, and transferred onto PVDF membranes (Bio-Rad, United States). After blocking with 10% non-fat milk solution, the membranes were incubated with specific primary antibodies such as IL-1β (Abcam, MA, United States; 1:1,000), Mincle (Bioss, China; 1:1,000), p-syk (Abcam; 1:500), and β-Actin (CST, MA, United States; 1:1,000) overnight at 4°C and subsequently incubated with HRP-conjugated secondary antibodies (Jackson, United States) for 2 h at room temperature. The protein bands were visualized using enhanced chemiluminescence reagents and captured using the ChemiDoc™ Touch Imaging System (Bio-Rad, United States). The intensity of the bands was quantified using ImageJ (raw blot images were provided in *Supplementary Material*).

### TdT-mediated dUTP nick-end labeling

Brain tissues were fixed in paraformaldehyde for 24 h, embedded in paraffin, and sectioned serially. After deparaffinization in xylene twice and hydration in 100%, 95%, 70%, 50%, and 0% gradient ethanol, the sections were subjected to antigen retrieval using citric acid buffer (pH 6.0) at 95–100C for 20 min. After cooling, the sections were incubated in the working solution containing the enzyme solution and labeling solution at a ratio of 1:10 at 37°C for 1 hour, in accordance with the manufacturer’s procedure. After washing with PBS three times, the sections were incubated with NeuN primary antibody (1:400) overnight at 4°C. After rinsing with PBS, the sections were incubated with secondary antibody conjugated with Alexa Fluor 568 for 2 h at room temperature, followed by DAPI staining for 30 min at room temperature. The images were captured using an Olympus fluorescent microscope and analyzed using ImageJ.

### Immunofluorescent staining

After deparaffinization, hydration, and antigen retrieval as mentioned above, brain tissue sections were blocked in 5% donkey serum at room temperature for 1 h and were then incubated with primary antibodies: Iba-1 (Abcam, 1:200), Mincle (Bioss, 1:200), NeuN (Abcam, 1:400), MAP2 (Abcam, 1:2000), and SAP130 (Abcam, 1:200) at 4°C overnight. After rinsing with PBS three times, the sections were incubated with secondary antibodies conjugated to Alexa flour 488 and 568 at room temperature for 1 h. After washing with PBS three times, the sections were incubated with DAPI for 5 min, rinsed with PBS, and mounted with prolonged mounting medium. The images were captured using an Olympus fluorescent microscope.

### Statistical analysis

Values were represented as the mean ± SEM. All statistical analyses were performed using GraphPad Prism (version 8.0). Normal distribution was assessed using the Shapiro–Wilk normality test. Homogeneity of variances was assessed using Levene’s test. For comparisons between two groups, statistical significance was evaluated using Student’s t-test for normally distributed data with equal variances, Welch’s t-test for normally distributed data with unequal variances, and the Mann–Whitney U test (unpaired) or Wilcoxon signed-rank test (paired) for non-normally distributed data. For comparisons among three or more groups, one-way ANOVA was applied for normally distributed data with equal variances, Welch’s ANOVA was applied for normally distributed data with unequal variances, and the Kruskal–Wallis test was applied for non-normally distributed data. When the overall test was significant, *post hoc* comparisons were performed using Tukey’s multiple comparisons test. For comparisons among grouped data that consist of two independent variables, two-way ANOVA was performed for normally distributed data, and when significant main effects or interactions were found, a *post hoc* test was performed using Sidak’s multiple comparisons test. Statistical significance was defined as *P < 0.05, **P < 0.01, and ***P < 0.001.

## Results

### Sevoflurane induced neuronal death and neuroinflammation in juvenile mice

**FIGURE 1:**
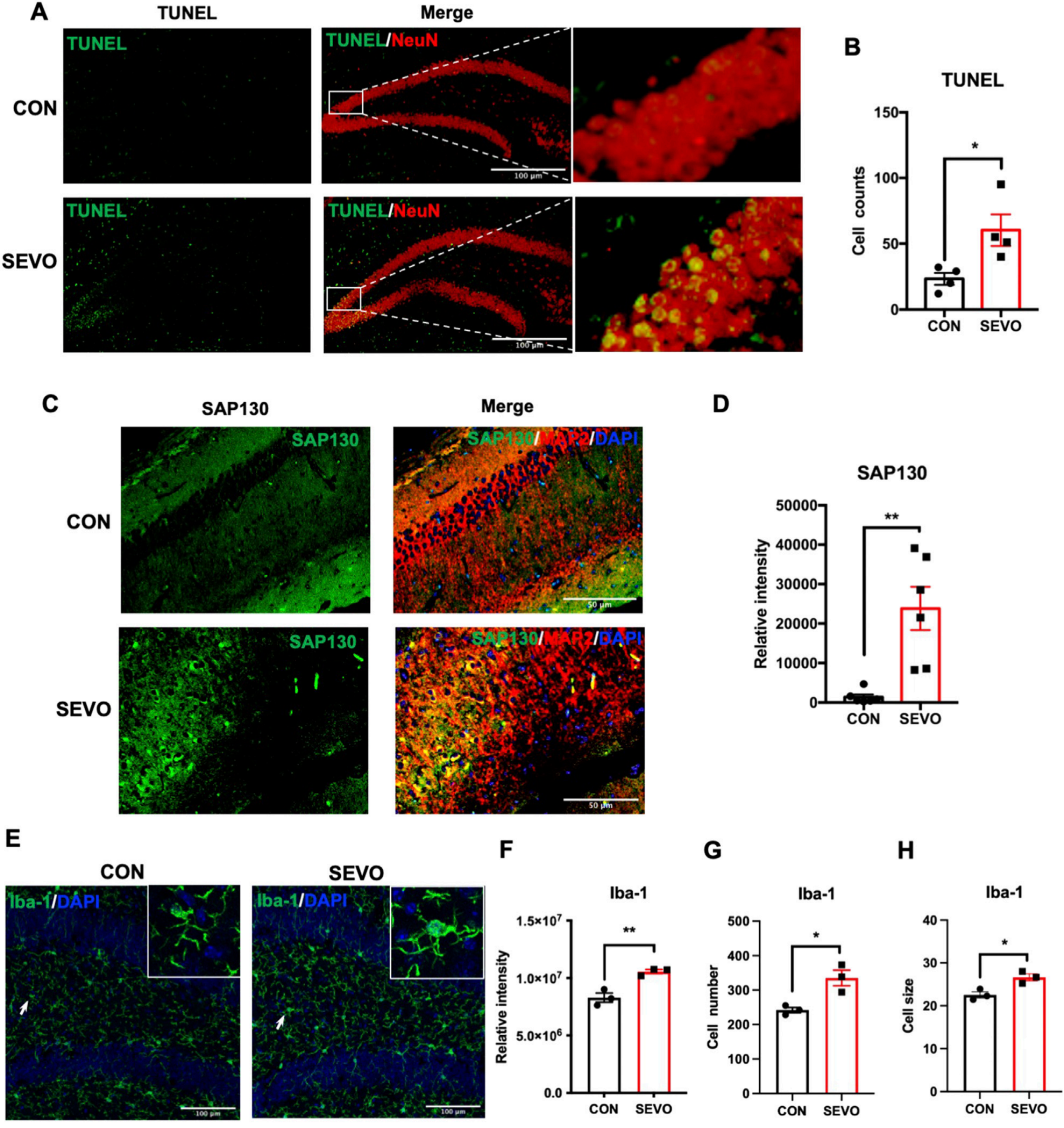
**(A)** Representative images showing TUNEL and NeuN staining and co-localization. **(B)** Histogram showing the cell number of TUNEL-positive cells in the hippocampus; n = 4 per group. **(C)** Representative images showing SAP130 and MAP2 staining and co-localization; n = 6 per group. **(D)** Histogram showing the quantification of SAP130 relative intensity. **(E)** Representative images showing Iba-1 staining. **(F)** Histogram showing the quantification of Iba-1 relative intensity. **(G)** Histogram showing the number of Iba-1-positive cells. **(H)** Histogram showing changes in cell size of Iba-1-positive cells; n = 3 per group. Student’s t-test; data are presented as the mean ± SEM; *P < 0.05 and **P < 0.01.

Sevoflurane has been shown to exert neurotoxic effects on the developing brain, which contributes to abnormal behavior or cognitive decline (McCann and Soriano, 2019; Liu et al., 2020; Urits et al., 2020). Sevoflurane exposure causes temporary cognitive dysfunction in children during the juvenile period. However, the underlying mechanism remains unclear. To address this question, we applied 2-h exposure of 3.5% sevoflurane to postnatal 21-day (P21) mice as a sevoflurane-exposed young mouse model and evaluated the neuronal death using the TUNEL assay. The results showed a higher number of TUNEL^+^ cells in the hippocampus of the SEVO group, and these cells were mainly co-localized with NeuN, indicating that sevoflurane induced neuronal death in juvenile mice [t (6) = 2.882; p = 0.028] (Figures 1A,B). SAP130, a type of DAMP, is mainly released from dead or dying cells and drives inflammatory responses by engaging with the Mincle receptor (Lv et al., 2021; Zhang et al., 2024a; He et al., 2015). Immunofluorescent staining revealed an upregulation of SAP130 immunoreactivity in the SEVO group [t (10) = 4.054; p = 0.0023], which was co-localized with the neuronal biomarker MAP2 (Figures 1C,D). Thus, sevoflurane-induced neuronal death was associated with neuronal SAP130 upregulation. Furthermore, sevoflurane exposure also led to microglial activation, as evidenced by increases in Iba-1 intensity [t (4) = 5.15; p = 0.0067], cell number [t (4) = 3.865; p = 0.0181], and cell size [t (4) = 3.735; p = 0.0202] of Iba-1-positive cells in the hippocampus (Figures 1E–H). These results indicate that SAP130 may be involved in sevoflurane-induced neuronal death and neuroinflammation in the developing hippocampus.

### SAP130 released by damaged neurons promoted microglial activation and exacerbated neuronal cell death after sevoflurane exposure

**FIGURE 2:**
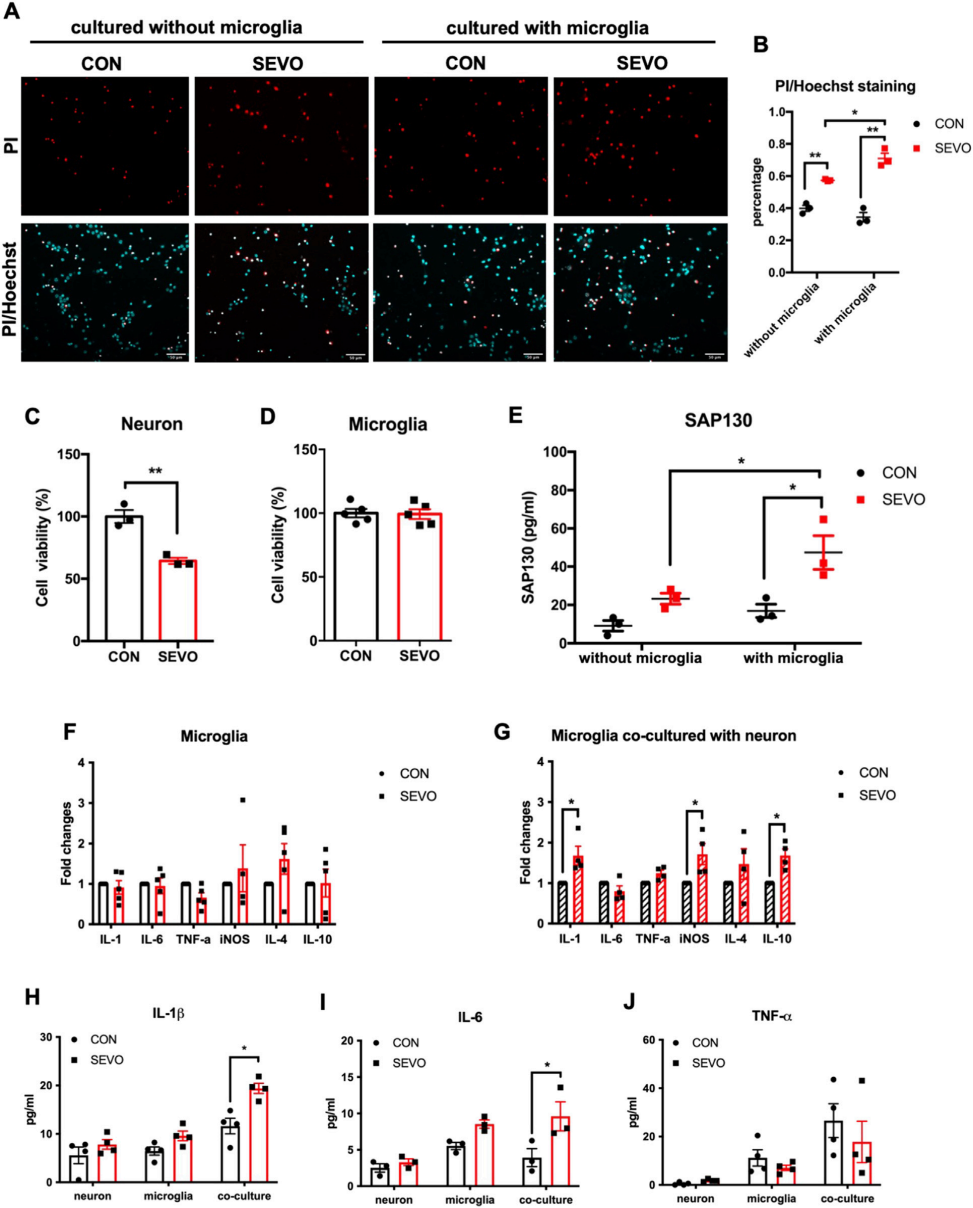
**(A)** Representative images of PI/Hoechst staining in primary neurons. **(B)** Histogram showing changes in the percentage of dead neurons after sevoflurane exposure; n = 3 per group. Two-way ANOVA followed by multiple comparisons test. **(C)** Histogram of CCK-8 assay showing cell viability of neurons after sevoflurane exposure; n = 3 per group. **(D)** Histogram of the CCK-8 assay showing cell viability of microglia after sevoflurane exposure; n = 5 per group; Student’s t tests. **(E)** Histogram showing the quantification of SAP130 in the cultured medium after sevoflurane exposure; two-way ANOVA followed by Sidak’s multiple comparisons test. **(F)** Histogram showing changes in cytokine mRNA levels in primary microglia; two-way ANOVA followed by Sidak’s multiple comparisons test. **(G)** Histogram showing changes in cytokine mRNA levels in primary microglia co-cultured with neurons. **(H)** Histogram showing changes in IL-1β protein levels in cultured medium; n = 6 per group. **(I)** Histogram showing changes in IL-6 protein levels in cultured medium; n = 3 per group. **(J)** Histogram showing changes in TNF-α protein levels in the cultured medium; n = 4 per group. Two-way ANOVA followed by Sidak’s multiple comparisons test. Data are presented as the mean ± SEM; *p < 0.05 and **p < 0.01.

Considering that sevoflurane exposure could activate microglia, which in turn impair neurons and cause cognitive dysfunction, we used a neuron–microglia co-culture model to investigate whether the existence of microglia influences neuron death after sevoflurane treatment. The results showed that sevoflurane exposure increased the percentage of PI-positive neurons, regardless of the presence of microglia. Simultaneously, neurons cultured with microglia had a higher percentage of PI-positive cells than those cultured without microglia after sevoflurane exposure [F (1, 8) = 132.3; p < 0.0001] (Figures 2A,B). We used the CCK-8 assay to examine the cell viability in neurons and microglia separately after sevoflurane treatment. The results showed that the cell viability of neurons was significantly decreased in the SEVO group [t (4) = 6.218; p = 0.0034] (Figure 2C), while no significant difference was found in microglia [t (8) = 0.1414; p = 0.8911] (Figure 2D). Since SAP130 is predominantly released from dead or dying cells, we next examined the secretion of SAP130 after sevoflurane exposure. The results indicated that SAP130 release was increased in the SEVO group compared to their counterparts, regardless of the presence of microglia (Figure 2E). Meanwhile, the level of SAP130 [F (1, 8) = 18.83; p = 0.0031] in the SEVO group co-cultured with microglia was even higher than that without microglia (Figure 2E). Thus, the alternation in secreted SAP130 was strongly associated with neuronal death under conditions of co-culture with microglia and exposure to sevoflurane, indicating that SAP130 secretion primarily derived from dead or dying neurons induced by sevoflurane.

We further applied sevoflurane treatment under the same conditions to primary microglia culture and neuron–microglia co-culture models to examine the impact of sevoflurane on microglial activation. Interestingly, 3.5% sevoflurane exposure did not induce the upregulation of cytokines in mono-cultured microglia [F (1, 23) = 0.5265; p = 0.4754] (Figure 2F), but microglia co-cultured with neurons showed transcriptional augmentation in IL-1β, iNOS, and IL-10 after sevoflurane treatment [F (1, 36) = 21.6; p < 0.0001] (Figure 2G). Moreover, IL-1β [F (1, 18) = 18.61; p = 0.0004] and IL-6 [F (1, 12) = 13.59; p = 0.0031] protein levels were increased in the neuron–microglia co-cultured medium after sevoflurane exposure, rather than in the neuron- or microglia-cultured medium (Figures 2H,I), while no significant change in TNF-α [F (1, 18) = 0.9849; p = 0.3342] was found in neurons, microglia, or the neuron–microglia co-cultured medium (Figure 2J). These observations indicated that SAP130 released from dead or dying neurons could facilitate microglial activation by promoting cytokine production, and activated microglia, in turn, aggravated sevoflurane-induced neuronal death.

### SAP130 promoted microglial activation by activating the Mincle/syk pathway after sevoflurane exposure

**FIGURE 3:**
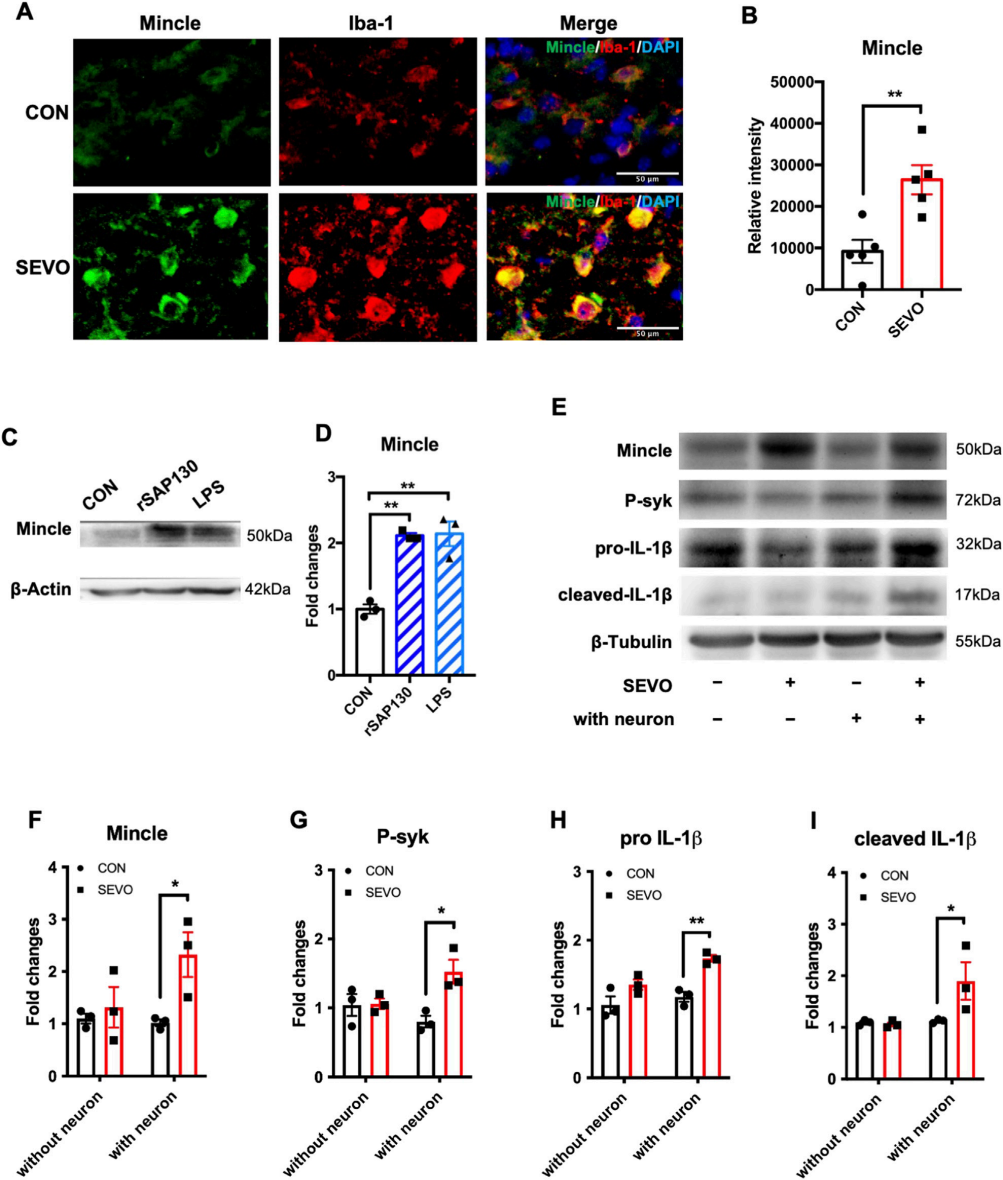
**(A)** Representative images of Mincle and Iba-1 co-staining in the hippocampus. **(B)** Histogram showing the quantification of Mincle relative intensity in the hippocampus; n = 5 per group. Student’s t-test. **(C)** Representative WB images of primary microglia treated using recombinant SAP130 or LPS. **(D)** Histogram showing Mincle expression in primary microglia; n = 3 per group. One-way ANOVA followed by Tukey’s multiple comparisons test. **(E)** Representative WB images of primary microglia after sevoflurane exposure or being co-cultured with primary neurons. **(F)** Histogram showing Mincle expression in primary microglia. **(G)** Histogram showing phosphorylated syk expression in primary microglia. **(H)** Histogram showing pro-IL-1β expression in primary microglia. **(I)** Histogram showing cleaved IL-1β expression in primary microglia. n = 3 per group; two-way ANOVA followed by Sidak’s multiple comparisons test. Data are presented as the mean ± SEM; *P < 0.05 and **P < 0.01.

Considering that sevoflurane exposure contributed to neuronal SAP130 release and triggered neuroinflammation and SAP130 could activate the Mincle signaling pathway, we hypothesized that SAP130 released from neurons triggered the Mincle/syk pathway and contributed to sevoflurane-induced neuroinflammation. To address this hypothesis, we investigated the change in Mincle and its co-localization in the sevoflurane-exposed hippocampus. We observed that Mincle was mainly co-localized with Iba-1-positive cells and significantly upregulated in the sevoflurane group [t (8) = 3.861; p = 0.0048] (Figures 3A,B). To determine whether Mincle upregulation depends on SAP130, we incubated primary microglia with recombinant SAP130 protein or LPS, which has been shown to induce Mincle expression in macrophages, served as a positive control (Sun et al., 2024; Tan et al., 2023). The results showed that both SAP130 protein and LPS treatment elevated Mincle expression in primary microglia [F (2, 6) = 30.96; p = 0.0007] (Figures 3C,D). In addition, we investigated the Mincle/syk pathway in the neuron–microglia co-cultured system. The increase in Mincle in mono-cultured microglia was found in the sevoflurane group, but it was not statistically significant. Sevoflurane did not affect the protein levels of phosphorylated syk, pro-IL-1β, or cleaved IL-1β, while it significantly elevated the protein levels of Mincle [F (1, 8) = 6.642, p = 0.0328], phosphorylated syk [F (1, 8) = 8.214; p = 0.0210], pro-IL-1β [F (1, 8) = 24.60; p = 0.0011], and cleaved IL-1β [F (1, 8) = 5.605; p = 0.0454] in microglia co-cultured with neurons (Figures 3E–I). These results suggested that SAP130 promoted microglial activation with IL-1β elevation by activating the Mincle pathway.

### Neutralization of SAP130 alleviated microglial activation and neuronal cell death by inhibiting the Mincle signaling pathway

**FIGURE 4:**
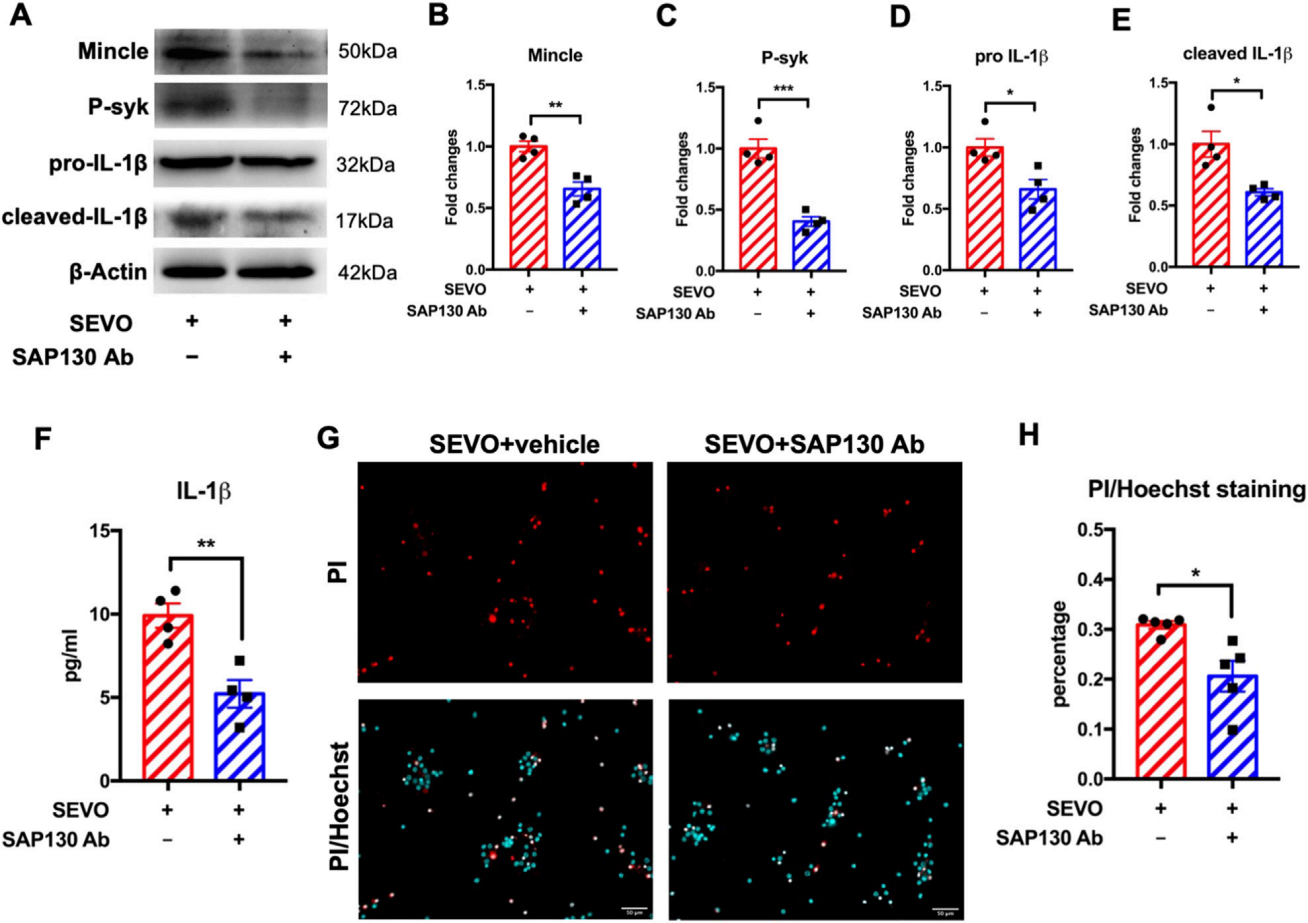
**(A)** Representative WB images of primary microglia treated with recombinant SAP130 and sevoflurane. **(B)** Histogram showing Mincle expression in primary microglia. **(C)** Histogram showing phosphorylated syk expression in primary microglia. **(D)** Histogram showing pro-IL-1β expression in primary microglia. **(E)** Histogram showing cleaved IL-1β expression in primary microglia; n = 4 per group. **(F)** Histogram showing the quantification of IL-1β in the cultured medium; n = 4 per group. **(G)** Representative images of PI/Hoechst staining in primary neurons. **(H)** Histogram showing changes in the percentage of dead neurons after recombinant SAP130 and sevoflurane treatment; n = 5 per group. Student’s t-test; data are presented as the mean ± SEM. *P < 0.05, **P < 0.01, and ***P < 0.001.

To further elucidate the role of SAP130 in Mincle pathway activation, we applied SAP130-neutralizing antibody in the neuron–microglia co-culture during sevoflurane exposure and detected the levels of Mincle pathway-related proteins in microglia using Western blot. We observed that SAP130 neutralization reduced microglial protein levels of Mincle [t (6) = 4.875; p = 0.0028], phosphorylated syk [t (6) = 6.909; p = 0.0005], pro-IL-1β [t (6) = 3.206; p = 0.0185], and cleaved IL-1β [t (6) = 3.627; p = 0.0110] (Figures 4A–E), consistent with the ELISA results showing a decrease in secreted IL-1β in the co-cultured medium [t (6) = 4.263; p = 0.0053]. These findings indicated that blockade of SAP130 attenuated microglial activation (Figure 4F). Since SAP130 is a mediator of microglial activation and activated microglia exacerbate neuronal death induced by sevoflurane, we further examined whether SAP130 blockade could protect neurons from sevoflurane-induced neurotoxicity. The results demonstrated that specific SAP130 antibody application reduced the percentage of dead neurons that were exposed to sevoflurane [t (8) = 3.230; p = 0.0121] (Figures 4G,H). These findings suggested that SAP130 blockage alleviated sevoflurane-induced neuronal death by inhibiting microglial activation via the Mincle/syk/IL-1β pathway.

### Piceatannol suppressed the activation of Mincle signaling pathway in microglia and attenuated neuronal cell death

**FIGURE 5:**
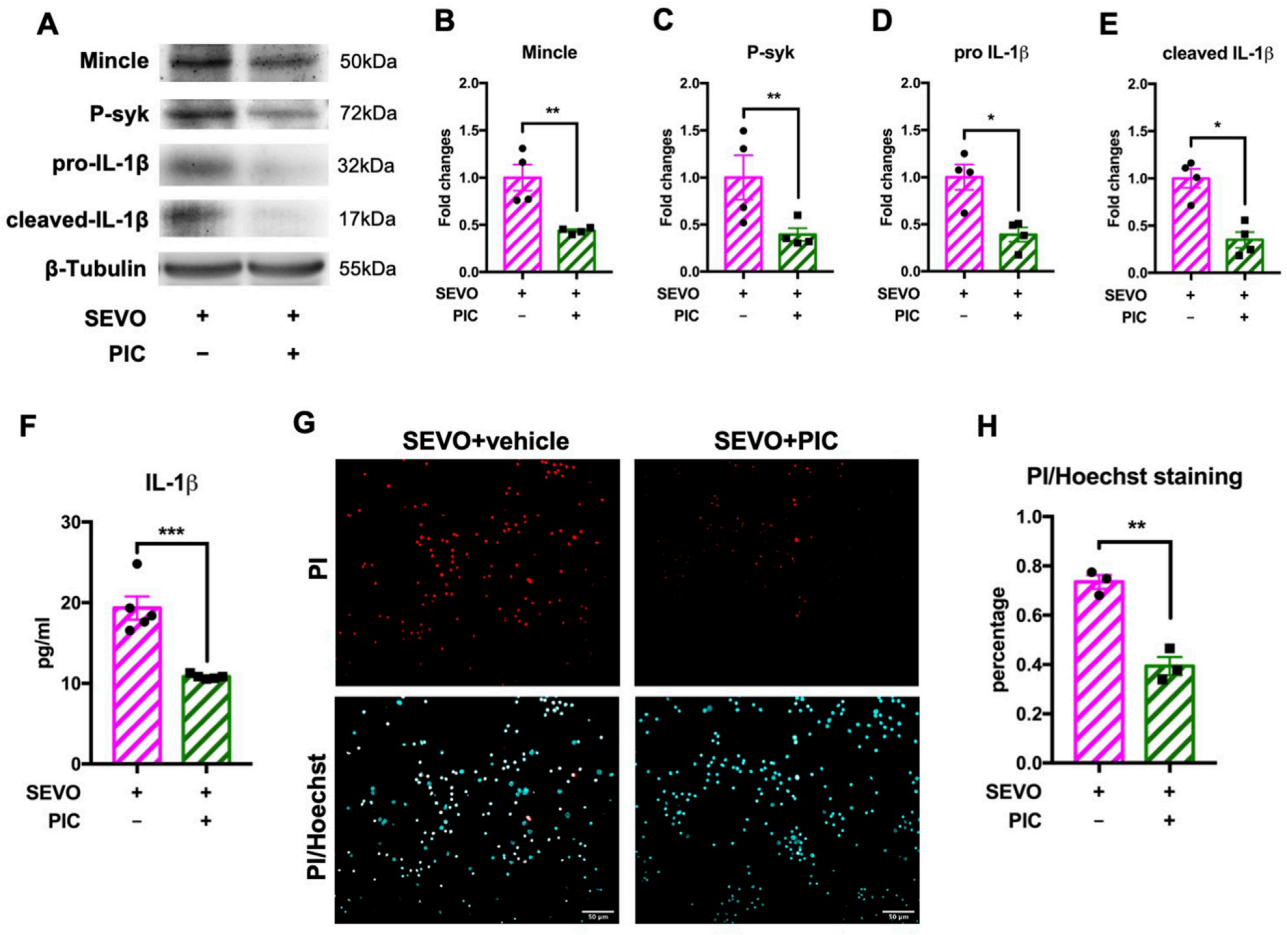
**(A)** Representative WB images of primary microglia treated with piceatannol and sevoflurane. **(B)** Histogram showing Mincle expression in primary microglia. **(C)** Histogram showing phosphorylated syk expression in primary microglia. **(D)** Histogram showing pro-IL-1β expression in primary microglia. **(E)** Histogram showing cleaved IL-1β expression in primary microglia; n = 4 per group. **(F)** Histogram showing the quantification of IL-1β in cultured medium; n = 5 per group. **(G)** Representative images of PI/Hoechst staining in primary neurons. **(H)** Histogram showing changes in the percentage of dead neurons after piceatannol treatment and sevoflurane exposure; n = 5 per group. Student’s t-test; data are presented as the mean ± SEM. *P < 0.05, **P < 0.01, and ***P < 0.001.

We further used piceatannol, a specific syk inhibitor, to determine the role of the Mincle/syk pathway in sevoflurane-induced microglial activation and neuronal death. We observed that piceatannol downregulated the expressions of Mincle [t (6) = 4.007; p = 0.0071], phosphorylated syk [t (6) = 2.461; p = 0.0491], pro-IL-1β [t (6) = 3.936; p = 0.0077], and cleaved IL-1β [t (6) = 4.943; p = 0.0026] in microglia (Figures 5A–E). Moreover, measurement of IL-1β in the cultured medium showed a significant decrease in the piceatannol treatment group (t (8) = 5.872, p = 0.0004), which was consistent with microglial IL-1β expression (Figure 5F). These changes were in line with the results after SAP130 neutralization. Then, we evaluated the impact of piceatannol treatment on neuronal death. The results showed that piceatannol significantly attenuated sevoflurane-induced neuronal death [t (4) = 7.284; p = 0.0019] (Figures 5G,H). These results demonstrated that the Mincle/syk/IL-1β pathway was involved in sevoflurane-induced microglial activation and neuronal death.

### Pharmacological inhibition of the Mincle pathway rescued sevoflurane-induced neuronal death in juvenile mice

**FIGURE 6:**
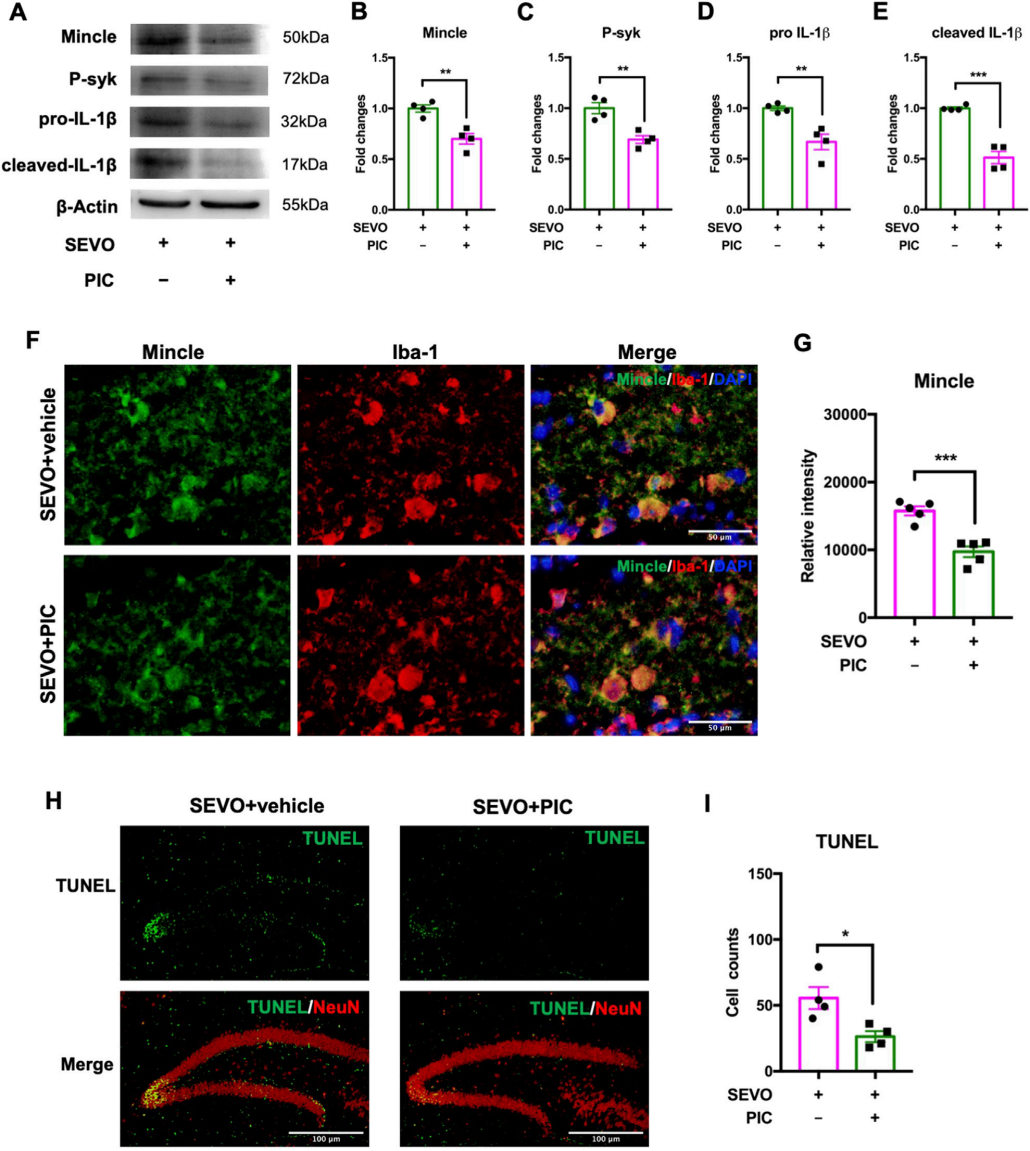
**(A)** Representative WB images of the hippocampus treated with piceatannol and sevoflurane. **(B)** Histogram showing Mincle expression in the hippocampus. **(C)** Histogram showing phosphorylated syk expression in the hippocampus. **(D)** Histogram showing pro-IL-1β expression in the hippocampus. **(E)** Histogram showing cleaved IL-1β expression in the hippocampus; n = 4 per group. **(F)** Representative images of Mincle and Iba-1 staining in the hippocampus. **(G)** Histogram showing the quantification of relative Mincle intensity in the hippocampus; n = 5 per group. **(H)** Representative images of TUNEL and NeuN staining in the hippocampus. **(I)** Histogram showing the cell number of TUNEL-positive cells after piceatannol treatment and sevoflurane exposure; n = 4 per group. Student’s t-test; data are presented as the mean ± SEM. *P < 0.05, **P < 0.01, and ***P < 0.001.

Considering that secreted SAP130 promoted microglial activation and aggravated neuronal death via the Mincle pathway in the neuron–microglia co-culture model, we further examined the protective effects of piceatannol on the developmental brain using the sevoflurane-exposed juvenile mouse model. The immunoblot results showed that piceatannol significantly downregulated the Mincle pathway-related proteins, including Mincle receptor [t (6) = 4.790; p = 0.0030], phosphorylated syk [t (6) = 4.668; p = 0.0034], pro-IL-1β [t (6) = 4.194; p = 0.0057], and cleaved IL-1β [t (6) = 7.892; p = 0.0002] in the sevoflurane-exposed hippocampus (Figures 6A–E). Similarly, the intensity of microglial Mincle was decreased in the piceatannol treatment group [t (8) = 5.891; p = 0.0004] (Figures 6F,G). Furthermore, there were less TUNEL-positive cells in the hippocampus after piceatannol treatment than their sevoflurane-only counterparts [t (6) = 3.139; p = 0.0201] (Figures 6H,I). These findings demonstrated that piceatannol alleviated sevoflurane-induced neuronal death in the developmental brain by inhibiting the Mincle pathway.

## Discussion

Our previous studies have demonstrated that prolonged exposure to inhaled anesthetics induces cognitive impairment in aged mice by enhancing neuroinflammation in the hippocampus (Wang et al., 2018). Although the neurotoxicity of inhaled anesthesia in the developing brain is controversial, experimental studies and clinical data focusing on the early postoperative period still implicate the neurotoxicity of general anesthetics by inducing cognitive abnormalities. Sevoflurane, as one of the most commonly used inhaled anesthetics in pediatric anesthesia, appears to be more likely to induce cognitive impairment in children and has been proven to cause neuronal cell death and neuroinflammation in rodent brains (Urits et al., 2020). In this study, we found that sevoflurane can induce neuronal cell death and microglial activation in the hippocampus of juvenile mice. Moreover, SAP130 released from dead neurons activated microglia, which, in turn, exacerbated neuronal cell death. Further investigation revealed that SAP130 mediated the interaction between neuronal cell death and microglial activation through the Mincle/syk pathway. Neutralizing SAP130 or suppressing syk successfully attenuated sevoflurane-caused neuronal cell death and microglial activation. Our study is among the first to elucidate the role of SAP130 in sevoflurane-induced neurotoxicity as the key regulator of the interaction between neuronal cell death and microglia activation by activating the Mincle/syk/IL-1β signaling pathway. Interference with SAP130 or the downstream Mincle pathway may represent a potential therapeutic strategy to ameliorate sevoflurane-induced neurotoxicity or even cognitive impairment.

Several studies have shown that neuroinflammation is a vital component in the pathogenesis of postoperative neurocognitive disorders, including anesthesia-related cognitive abnormalities (Zhu et al., 2017; Alam et al., 2018; Liu et al., 2023b; Luo et al., 2019; Huang et al., 2021). Microglia, as resident immune cells in the central nervous system, play critical roles in mediating neuroinflammation and the progression of neurological diseases characterized by neuroinflammation. Previous studies have shown that general anti-inflammatory treatments preserved postoperative cognitive functions by reversing microglial inflammatory responses or the M1 phenotype (Wang et al., 2016; Liang et al., 2023; Oberman et al., 2021). Regarding sevoflurane-related cognitive impairment, activated microglia served as a key contributor to neurotoxicity in either developmental or aged rodents via different molecular mechanisms (Huang et al., 2021; Xu et al., 2023a; Gao et al., 2024; Zhang et al., 2024b). Recent studies showed that sevoflurane-activated microglia phagocytosed synapses via the complement pathway (Xu et al., 2023a; Wang et al., 2024). Another study showed that sevoflurane induced the activation of the BV2 microglial cell line, which further induced apoptosis in HT-22 neuronal cells (Dai et al., 2022). Furthermore, neuron-released exosomes have been found to transfer lncRNAs that mediate microglial M1 polarization and contribute sevoflurane-induced neurotoxicity (Xu et al., 2024). These observations prove that the activation of microglia contributes to sevoflurane-induced neurotoxicity. In this study, we found that sevoflurane-induced neuronal cell death was aggravated in the presence of microglia that were simultaneously activated by sevoflurane. These findings indicate the involvement of microglia in sevoflurane-induced neurotoxicity, which aligns with previous research. Moreover, a key observation in this study is that microglial activation required SAP130 released from damaged or dead neurons, instead of being activated by sevoflurane alone, indicating that SAP130 is necessary for sevoflurane-triggered microglial activation. Given that activated microglia caused more neuronal death and that SAP130 release further reinforced microglial activation, these findings underscore SAP130’s role as a pivotal mediator in the crosstalk between neuronal cell damage and microglial activation, providing insights into the mechanism of sevoflurane-induced neurotoxicity.

The Mincle receptor, a c-type lectin receptor, is widely expressed in antigen-presenting cells and acts as a pattern-recognition receptor that can recognize pathogen-associated molecular patterns, like bacteria or fungi, to induce immune responses against microbial infections. Mincle can also identify danger-associated molecular patterns like SAP130 or β-glucosylceramides, which further initiate inflammation and accelerate tissue damage (Huang et al., 2023; Zou et al., 2024; Drouin et al., 2020). It has been reported that SAP130 released by damaged or dead tubular cells increases the expression of Mincle and its downstream molecules to promote macrophage activation, which, in turn, aggravates tubular epithelial cell death in acute kidney injury animal models (Lv et al., 2021; Zhang et al., 2024a). Similarly, the upregulation of Mincle and SAP130 in the liver was observed in both mice and humans with autoimmune hepatitis, which was attenuated by Mincle blockage or deletion but aggravated by Mincle ligation (Greco et al., 2016). Recent research also implicated SAP130 as a key mediator in the crosstalk between hepatic ferroptosis and macrophage activation in perfluorooctane sulfonate (PFOS)-induced liver injury (Li et al., 2024). In the central nervous system, it has been found that the SAP130/Mincle axis is involved in promoting neuroinflammation in various brain injury rodent models, including ischemic stroke, traumatic brain injury, and subarachnoid hemorrhage (de Rivero Vaccari et al., 2015; He et al., 2015; Suzuki et al., 2013). Neutralization of Mincle by specific antibody or albumin attenuated neuronal cell death and improved neurological functions in subarachnoid hemorrhage (Xie et al., 2017; He et al., 2015). Consistently, the SAP130/Mincle pathway was activated in multiple sclerosis, and Mincle silencing or SAP130 neutralization has protective effects by suppressing neuroinflammation (N'Diaye et al., 2020). In this study, we observed that SAP130 upregulation and microglial Mincle expression were induced by sevoflurane treatment in the hippocampus of juvenile mice and primary neuron–microglia co-culture systems. Interestingly, both sevoflurane and recombinant SAP130 can directly induce microglial Mincle expression, but sevoflurane could not upregulate microglial cytokine expression in the absence of SAP130. Furthermore, SAP130 facilitated sevoflurane-induced microglial activation with higher IL-1β expression, which, in turn, aggravated neuronal cell death. These results indicated that sevoflurane exposure could upregulate Mincle expression directly, whereas microglial IL-1β upregulation required SAP130. Both neutralization of SAP130 and blockade of syk significantly inhibited the expression of Mincle and its downstream proteins, which further alleviated microglial IL-1β expression and neuronal cell death. The reduction in Mincle after neutralization of SAP130 or syk blockade implied that SAP130 not only affected cytokine production but also enhanced Mincle expression to augment the activation of the Mincle axis and neuroinflammatory responses. These observations elucidated the molecular mechanism of the SAP130/Mincle axis in the pathogenesis of sevoflurane-induced neurotoxicity and suggested the Mincle pathway as a potential therapeutic target in sevoflurane-related neurotoxicity, neuroinflammation, and even cognitive disorders.

However, there are some limitations to this study as we only used juvenile male mice and applied the transwell chamber for the neuron–microglia co-culture system. We acknowledge that sevoflurane-induced neurotoxicity is not gender- or age-specific and that the elderly brain is also vulnerable and sensitive to sevoflurane exposure. Although the data support our hypothesis, further studies are required in both sexes and aged animals. In addition to cytokine production, activated microglia can phagocytose synapses that further impair cognition (Wang et al., 2024; Xu et al., 2023a). However, we only demonstrated the adverse effects of microglial activation on neuronal viability by primarily releasing IL-1β. We will investigate the change in other microglial inflammatory factors, like C1q, and microglial phagocytosis and their effects on neuronal viability and functions in the future. Moreover, recent studies suggest that reactive astrocytes are implicated in neuroinflammation and the progression of neurological diseases (Liddelow and Barres, 2017; Liddelow et al., 2017; Lawrence et al., 2023). It should be worthwhile to further investigate the role of reactive astrocytes in sevoflurane-induced neurotoxicity.

## Conclusion

Taken together, SAP130 released from damaged neurons is a crucial molecule that initiates sevoflurane-induced microglial activation, and activated microglia aggravate neuronal cell death after sevoflurane exposure. The SAP130/Mincle axis plays a significant role in the crosstalk between neuronal cell death and microglial activation, which contributes to the development of sevoflurane-induced neurotoxicity. It further supports that targeting the SAP130/Mincle axis could be the potential strategy to ameliorate sevoflurane-induced neurotoxicity.

## Data Availability

The original contributions presented in the study are included in the article/supplementary material; further inquiries can be directed to the corresponding authors.
